# Transcriptome Analysis Provides Insights into the Markers of Resting and LPS-Activated Macrophages in Grass Carp (*Ctenopharyngodon idella*)

**DOI:** 10.3390/ijms19113562

**Published:** 2018-11-12

**Authors:** Yazhen Hu, Xiaolei Wei, Zhiwei Liao, Yu Gao, Xiaoling Liu, Jianguo Su, Gailing Yuan

**Affiliations:** 1 Department of Aquatic Animal Medicine, College of Fisheries, Huazhong Agricultural University, Wuhan 430070, China; YazhenHu_941017@163.com (Y.H.); weixiaolei1205@163.com (X.W.); liaozhiwei1991@163.com (Z.L.); liuxl@mail.hzau.edu.cn (X.L.); sujianguo@mail.hzau.edu.cn (J.S.); 2 Hubei Engineering Technology Research Center for Aquatic Animal Disease Control and Prevention, Wuhan 430070, China; 3 College of Animal Science and Technology, Yunnan Agricultural University, Kunming 650201, China; gaoyu@ynau.edu.cn

**Keywords:** chemokine, *Ctenopharyngodon idella*, leukocyte differentiation antigen, lipopolysaccharide, macrophage

## Abstract

Macrophages are very versatile immune cells, with the characteristics of a proinflammatory phenotype in response to pathogen-associated molecular patterns. However, the specific activation marker genes of macrophages have not been systematically investigated in teleosts. In this work, leukocytes (WBC) were isolated using the Percoll gradient method. Macrophages were enriched by the adherent culture of WBC, then stimulated with lipopolysaccharide (LPS). Macrophages were identified by morphological features, functional activity and authorized cytokine expression. Subsequently, we collected samples, constructed and sequenced transcriptomic libraries including WBC, resting macrophage (Mø) and activated macrophage (M(LPS)) groups. We gained a total of 20.36 Gb of clean data including 149.24 million reads with an average length of 146 bp. Transcriptome analysis showed 708 differential genes between WBC and Mø, 83 differentially expressed genes between Mø and M(LPS). Combined with RT-qPCR, we proposed that four novel cell surface marker genes (*CD22*-like, *CD63*, *CD48* and *CD276*) and two chemokines (*CXCL*-like and *CCL39.3*) would be emerging potential marker genes of macrophage in grass carp. Furthermore, *CD69*, *CD180*, *CD27*, *XCL32a.2* and *CXCL8a* genes can be used as marker genes to confirm whether macrophages are activated. Transcriptome profiling reveals novel molecules associated with macrophages in *C. Idella*, which may represent a potential target for macrophages activation.

## 1. Introduction

The innate immune response of early vertebrates, such as bony fishes, plays a central role in host defense against infectious diseases and one of the most important effector cells for innate immunity is the macrophage [1]. In mammals, immune system cells originate from the hematopoietic stem cells in the bone marrow and perform functions encompassing host defense and tissue homeostasis [2,3]. However, the primary hematopoietic organ in the fish is the kidney [4,5]. Similar to mammals, macrophages in teleosts provide both an immediate defense against foreign agents and assistance in triggering of the adaptive immune response [6].

Recent research has shown that the initial trigger of macrophage polarization in teleosts fish could rely solely on the recognition of microbial/parasitic infection or danger signals in innate immunity and inflammation, enabling the polarization into M1 macrophages or M2 macrophages; this is also defined as innate-activated [7,8]. Notably, macrophages are very versatile immune cells, with the characteristics of a proinflammatory phenotype in response to pathogen-associated molecular patterns (PAMPs) [9]. LPS, serving as the best-studied microbial stimuli, can activate macrophages via a large array of pattern recognition receptors (PRRs) [10]. Researchers have shown that pro-inflammatory macrophages have an IL-12^high^, IL-23^high^, IL-10^low^ phenotype and are proficient producers of effector molecules for reactive oxygen, nitrogen intermediates, inflammatory cytokines (IL-1β, IL-6, TNF-α), acting as inducer and effector cells in polarized Th1 responses, and mediate resistance against intracellular parasites and tumors [11,12,13]. In mammals, the toll-like receptor 4 (TLR 4) can detect LPS leading to the secretion of pro-inflammatory cytokines, chemokines, antimicrobial peptides and other inflammatory mediators by the TLR4 complex. A typical feature of a mammalian immune response to the exposure to LPS is the intensive induction of numerous nuclear factor-kappa B (NF-κB) and IFN-γ responses mediated by the TLR4 signaling pathway [14,15]. However, the detailed functions and regulatory mechanisms of a macrophage in teleost is different from mammals. For example, teleost macrophages express novel cytokines to regulate inflammatory reactions and are poorly activated by TNF [6]. Herein, in this study, we chose LPS as a stimulus to study the activation of macrophages.

Macrophages are a group of heterogeneous immune cells that play a key role in the inflammatory response, which is caused by pathogenic microorganisms, tissue damage and the digestion process. Head kidney-derived macrophages in teleosts show functional polarization upon differential stimulation. M1 macrophages have a high antigen-presenting capacity and usually secrete a large number of related cytokines to kill pathogens and contribute to the inflammatory response [16]. In contrast, M2 macrophages play an important role in the reparative phase of inflammation [17,18], promoting tissue repair [19] and resisting parasitic infections [20,21]. Different signals can activate macrophages to change its own morphological and physiological characteristics, showing significant plasticity to deal with the external environment [9,22]. This characteristic has provided a therapeutic target whereby macrophages are encouraged to switch functionally from proinflammatory to anti-inflammatory [23,24]. However, the specific activation markers of macrophages have not been clearly elucidated in teleosts.

Additionally, the expression of cell surface markers, cytokine receptors and hematopoietic-related transcription factors can be used to determine the developmental stage of the cell [25,26]. In this study, to preserve clarity, Mø and M(LPS) were used to describe the resting and activated macrophages [27]. We firstly reported the transcriptional profile analysis on the *Ctenopharyngodon idella (C. idella)* Mø and M(LPS), describing distinct molecular signatures, which shed new light on these processes and revealed new candidate markers. This dataset served as a rich resource for identifying putative markers of *C. idella*. As a follow up approach, quantitative real-time PCR (qRT-PCR) was used to examine a panel of transcripts to verify the reproducibility of the gene expression changes from multiple groups.

## 2. Results

### 2.1. Enrichment and Morphological Identification of Head Kidney Primary Cells

**Figure 1:**
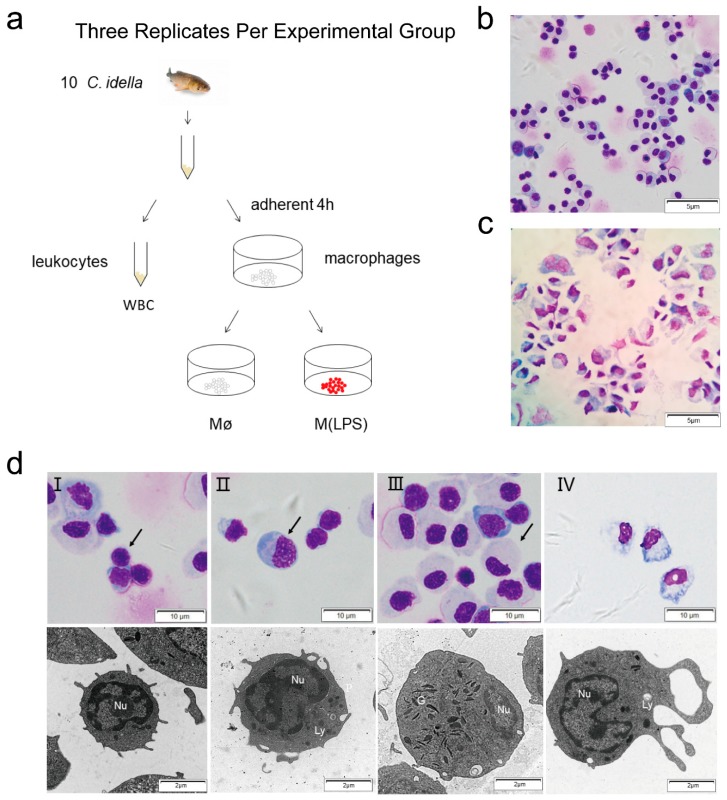
Leukocyte and macrophage isolation and identification. (**a**) Protocol for the preparation of leukocytes (WBC), macrophage (Mø) and M(LPS). The image of a fish in this picture was taken from Wikimedia Commons: https://commons.wikimedia.org/wiki/Main. (**b**) Low-magnification image analysis of Giemsa stain. WBCs (including lymphocytes, monocytes, neutrophils) were stained with Giemsa staining. (**c**) Macrophages were stained with Giemsa staining. (**d**) Lymphocytes (I), monocytes (II), neutrophils (III) and macrophages (IV) were examined under optical microscopy (OM) and transmission electron microscopy (TEM), the OM (scale bar = 10 μm) and TEM (scale bar = 2 μm), Nu = nucleus, Ly = lysosomes, G = specific granules.

Leukocytes and macrophages were obtained from *C. idella* head-kidney, this sampling process is shown in (Figure 1a). Leukocytes or macrophages were observed with Giemsa staining. Lymphocytes, monocytes and neutrophils were identified in leukocyte subpopulations (Figure 1b). Within 4 h, many of the leukocytes had adhered to the surface of microplate wells and the cells exhibited a characteristic irregular morphology. Adherent cells were identified as macrophages (Figure 1c). Electron microscopy was used to identify the subcellular morphology of leukocytes and adherent macrophages: Lymphocytes (3–5 μm) were identified by their characteristic large nuclei (Figure 1d, I), monocytes (10–15 μm) were characterized by a high cytoplasmic-to-nuclear ratio and small clear vacuoles in the cytoplasm (Figure 1d, II), neutrophils (8–15 μm) were distinguished by clear cytoplasm and segmented nuclei (Figure 1d, III). Macrophages had the following features: 10–20 μm in diameter, nucleus typically 5–7 μm in diameter, a large number of vacuoles in the cytoplasm and lysosomes tended to form multiple pseudopods (Figure 1d, IV) [28].

### 2.2. Survey on the Proposed Macrophage Markers Expression and the Respiratory Burst Activity

**Figure 2:**
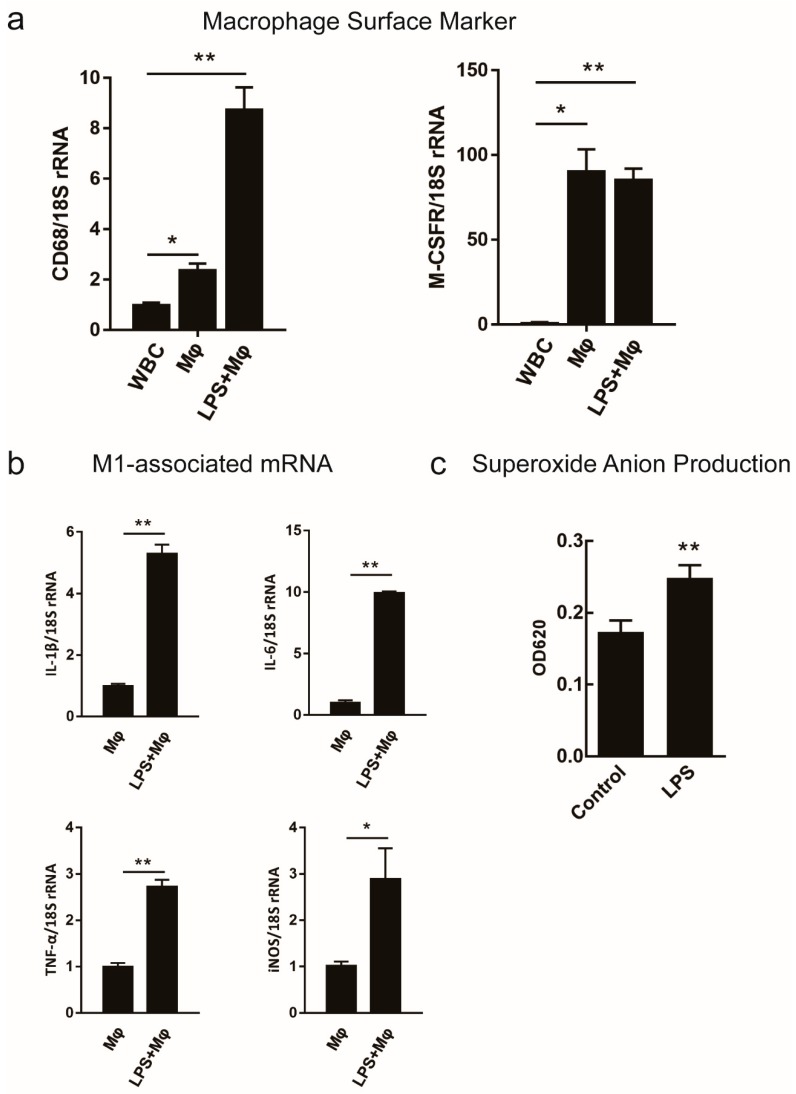
Expression of classical macrophage markers and detection of macrophage functional activity. A Comparison with WBC, qRT-PCR was used to determine the changes in the expression of each indicated transcript. (**a**) *CD68* and *M-CSFR* as the classical surface markers of macrophage were determined in Mø and M(LPS). (**b**) Proinflammatory factor levels of *IL-1β*, *IL-6*, *iNOS* (which elicited the production of reactive nitrogen), and TNF-α were determined in macrophage culture in vitro, stimulated with LPS (5 μg/mL) or without stimulation. (**c**) O^2−^ was measured by the reduction of nitroblue tetrazolium after the macrophages were incubated with 5 μg/mL of LPS or medium alone for 6 h. Data are expressed as mean ± SD (*n* = 3). * *P* < 0.05; ** *P* < 0.01.

Transcripts used as surface markers of macrophages should have a higher expression level in Mø and activated macrophages (M(LPS)) and transcripts used as markers of polarized macrophages should change expression in response to LPS. We evaluated the expression of transcripts that encoded two proposed resting markers in resting and LPS-activated macrophages [29,30], and four pro-inflammatory cytokines in LPS-activated macrophages by qRT-PCR [31]. Higher levels of *CD68* and *M-CSFR* were expressed on Mø and M(LPS) compared to WBC (Figure 2a). We observed an increase in transcription levels of *IL-1β*, *IL-6*, *TNF-α*, and *iNOS*, which were associated with macrophage activation of LPS but the effect was not significant (Figure 2b). Three independent biological replicate experiments showed that the expression of previously proposed markers showed the same trend (Supplementary Figure S1). It has been reported that LPS can modulate a macrophage into the M1-type and enhance phagocytosis and respiratory burst ability. Additionally, the content of the superoxide anion was used as an indicator of respiratory burst activity. Remarkably, as shown in Figure 2c, the incubation with LPS was able to elicit superoxide anion production in *C. idella* head-kidney macrophages (** *P* < 0.01). These results demonstrated that macrophages were efficiently isolated from *C. idella* head-kidney and slightly activated by LPS. Samples collected and pooled from three independent experiments produced the same trend by qRT-PCR (Supplementary Figure S2). These observations revealed that a systematic attempt was necessary in order to identify reliable markers whose expression was either up- or down-regulated in *C. idella* macrophages.

### 2.3. Characteristics of the Plastid Genomes

By Illumina HiSeq 2 × 150 bp pair-end sequence technology, a total of 149,241,970 clean reads (20.36 Gb of data bulk) with an average length of 146 bp were generated from the three libraries (WBC, Mø, and (M(LPS)). Subsequently, Hisat 2 (v2.0.1) was used to map clean reads to the *C. idella* genome references and the statistics of the alignment results were presented for each reference. The sequence data of the three transcriptomes (WBC, Mø, and M(LPS)) were able to be mapped to the draft genome, and their mapping coverage was 85.16, 85.73 and 86.86% respectively. The detailed assembly results (Table 1) indicate a good coverage of the assembled transcripts by the sequencing read.

**Figure 3:**
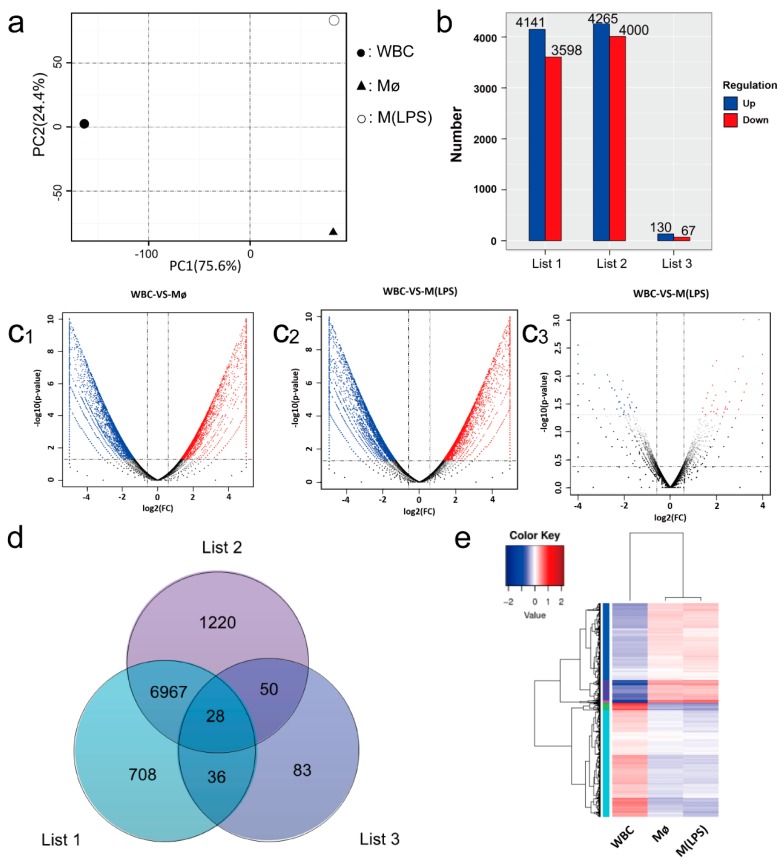
Transcriptome sequencing, assembly and analysis. (**a**) Principal Component Analysis (PCA) of the transcriptome expressed in WBC, Mø and M(LPS). PCA was carried on all genes under investigation to determine the expression trends within the data set. (**b**) The number of upregulated and down-regulated transcripts within each group. List 1 contains the up-regulated and down-regulated transcripts in Mø, compared to WBC; list 2 contains the up-regulated and down-regulated transcripts in M(LPS), compared to WBC, and list 3 contains the up-regulated and down-regulated transcripts in M(LPS), compared to Mø. (**c**) Volcano plots were applied to the significantly differentially expressed genes from three sets of samples. The absolute value of log2 ratio ≥ 1 and *P* ≤ 0.05 were used as the threshold to judge the significance of differentially Expressed Genes (DEGs). (**d**) Venn diagram that describes overlaps among differently regulated transcripts within each group. List 1 contains the up-regulated and down-regulated transcripts in Mø, compared to WBC; list 2 contains the up-regulated and down-regulated transcripts in M(LPS), compared to WBC, and list 3 contains the up-regulated and down-regulated transcripts in M(LPS), compared to Mø. (**e**) Two-dimensional hierarchical clustering was performed on the clusters of differentially expressed genes.

For the gene expression analysis, the number of expressed sequences was calculated and normalized to the number of reads per Kb per million reads (RPKM). The distributions of absolute expression (RPKM) are shown in box plots (Supplementary Figure S3). A principal component analysis (PCA) was applied to identify the factors that explained the most important variations among three datasets. PCA revealed that WBC samples were significantly different from the Mø samples in terms of the total variance, having a smaller change in the M(LPS) samples (Figure 3a). In general, PCA revealed that the gene expression profiles associated with Mø and M(LPS) were more closely related to each other than to WBC.

In order to determine the degree of variation in the responses among the three scorers, the transcriptome data were comparatively studied. The absolute value of log2 ratio  ≥  1 and *P* ≤ 0.05 were used as the threshold to determine the difference in gene expression. Compared with WBC, 35,984,000 up-regulated transcripts, and 41,414,265 down regulated transcripts were selected from Mø and M(LPS), respectively. During macrophage activation by LPS stimulus, there were 67 up-regulated transcripts and 130 down-regulated transcripts (Figure 3b). Among the over-expressed transcripts, Mø and M(LPS) showed a larger number of highly regulated transcripts relative to WBC (Figure 3(c_1_,c_2_)). In contrast, the results showed a small but significant difference in expression levels (Figure 3(c_3_)), compared with Mø and M (LPS). A comparative transcriptome analysis showed that 708 transcripts were uniquely different in WBC and Mø, 83 transcripts were uniquely different in Mø and M(LPS) (Figure 3d). The spectrum was evaluated and the dendrogram indicates the gene expression profiles of these three types of cells was summarized in the heat map. These results show that the transcript levels of WBC were significantly different from Mø and M(LPS), while the latter two were placed in a similar hierarchy (Figure 3e).

### 2.4. Identification of GO and KEGG Enrichment Analysis

**Figure 4:**
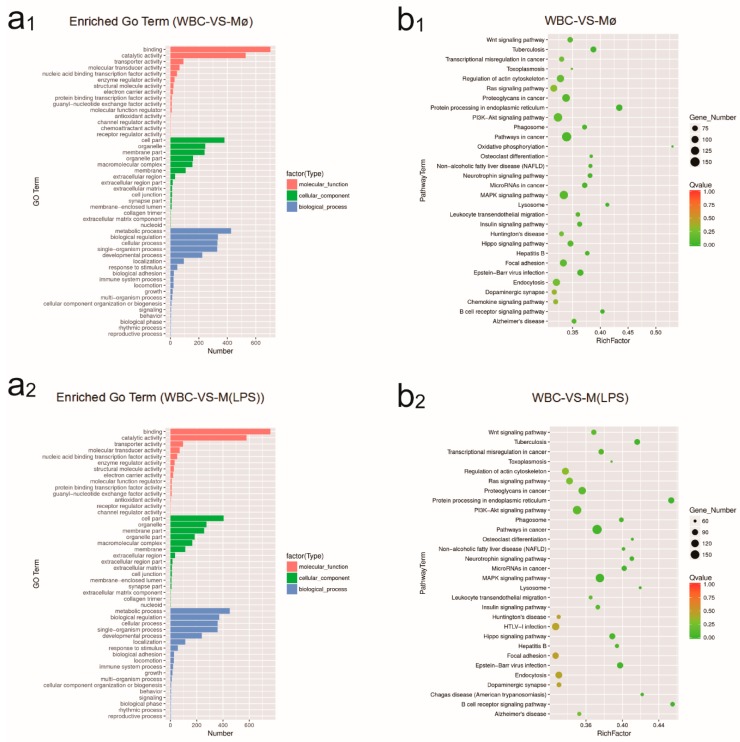

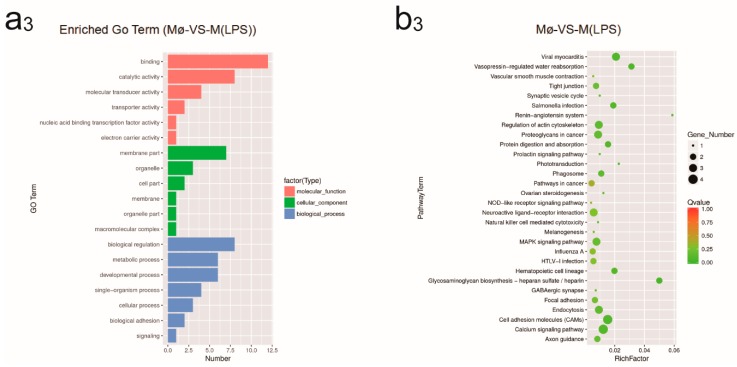
The analysis of DEGs annotation. (**a**) The number of differentially expressed gene annotations obtained from the three databases. Differentially expressed gene annotation hits from the Gene Ontology (GO) databases. Differentially expressed gene annotations obtained between WBC and Mø (**a_1_**), WBC and M(LPS) (**a_2_**), Mø and M(LPS) (**a_3_**). (**b**) Top 30 statistics of pathway enrichment between three sets of samples. In this scatter plot, the rich factor was the ratio of differentially expressed gene numbers annotated in this pathway term to all gene numbers annotated in this pathway term. Pathway enrichment between WBC and Mø (**b_1_**), WBC and M(LPS) (**b_2_**), Mø and M(LPS) (**b_3_**).

Gene Ontology (GO) analysis fell into three major categories: biological processes, cellular components, and molecular function. A comparison between Mø and M(LPS) showed a statistically significant difference relative to WBC. For example, in the category of “molecular function”, binding and catalytic activity were the prevailing terms. Meanwhile, membrane parts and cell parts were dominant groups in the “cellular component function” category. Additionally, in the “biological processes” category, genes involved in cellular and metabolic processes were most abundant (Figure 4(a_1_,a_2_)). Especially during macrophage activation, as per the “biological process” category, dominant subcategories were the cellular process and biological regulation (Figure 4(a_3_)).

KEGG, a bioinformatics resource, which records networks of molecular interactions in the cells and their variants specific to particular organisms. [32]. In this report, we have displayed the top 30 pathway terms enriched as the following. Altogether, with leukocytes as the control, there were 2245 DEGs for Mø assigned to 43 different pathways (Figure 4(b_1_)) and 2420 DEGs for M(LPS) assigned to 51 different pathways (Figure 4(b_2_)). During macrophage activation, a transcriptome analysis revealed that the differentially expressed genes were enriched in metabolism pathways such as glycosaminoglycan biosynthesis-heparan sulfate/heparin, protein digestion and absorption pathways. Particularly noteworthy was that transcriptome analysis was enriched in the calcium signaling pathway (Figure 4(b_3_)).

### 2.5. Evaluating the Use of CD Molecule Transcripts as Macrophages Markers

**Figure 5:**
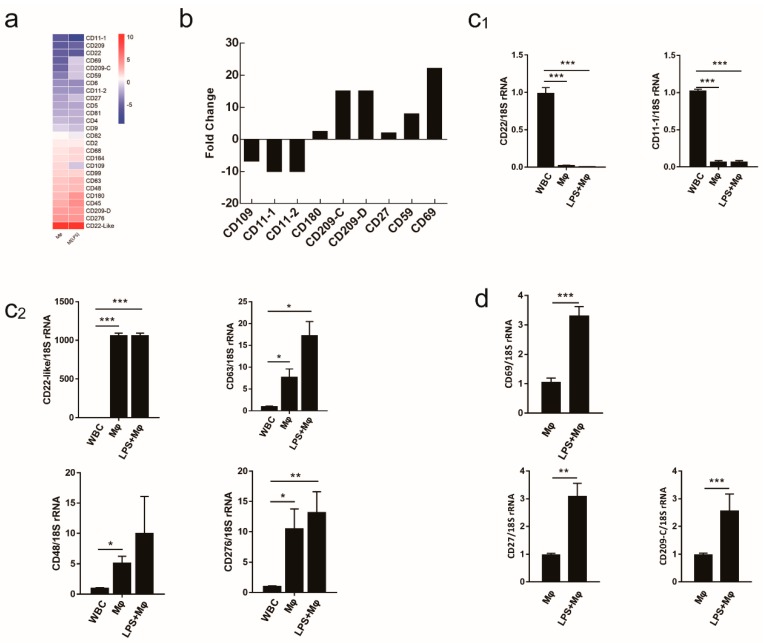
Evaluation of cluster of differentiation (CD) molecules as macrophage markers. (**a**) Using WBC as a control, all CD molecules were selected from a set of regulated transcripts and sorted according to average expression level changes. (**b**) CD molecules were further screened and sorted according to average expression level changes in response to the macrophage-activation treatment conditions. (**c**) The mRNA expression levels of CD molecules in Mø and M(LPS) comparing to WBC were tested by qRT-PCR. (**c_1_**) CD molecules have low expression in Mø and M(LPS) comparing to WBC. (**c_2_**) Compared to WBC, CD molecules have higher expression levels in Mø and M(LPS). (**d**) The fold changes of mRNA expression in M(LPS) relative to Mø. Data are expressed as mean ± SD (*n* = 3). * *P* < 0.05; ** *P* < 0.01; *** *P* < 0.001.

To investigate the expression of cluster of differentiation (CD) molecules on the surface of macrophages, an in-depth analysis of the transcriptome data revealed that macrophages (Mø and M(LPS)) showed significant differences in CD molecule expression compared to WBC (Figure 5a and Supplementary Dataset 1). Macrophage activation markers would ideally have large expression changes in the activated macrophage type. Therefore, the distinct expression signatures of CD molecules from M(LPS) were observed in the current study to identify the activation markers. Many putative activation markers were identified in macrophages activated with LPS (Figure 5b and Supplementary Dataset 1).

Since donor-to-donor variability among the *C. idella* head-kidney primary cells response was an issue of concern, the expression for many transcripts was determined in samples derived from the RNA-seq experiment and two additional groups whose *C. idella* head-kidney primary cells were isolated and cultured under the same conditions by qRT-PCR. qRT-PCR analysis of RNA samples from RNA-seq experiment showed that *CD22*-like, *CD63*, *CD48* and *CD276* were all expressed in Mø and M(LPS) (Figure 5c). It was also worth noting that M(LPS) expressed *CD69*, *CD180*, *CD27* and *CD209* antigen-like protein C (*CD209-C*) (Figure 5d). Additionally, samples collected and pooled from three independent experiments showed the same trend (Supplementary Figure S4).

### 2.6. Evaluating the Use of Chemokine Transcripts as Macrophage Markers

**Figure 6:**
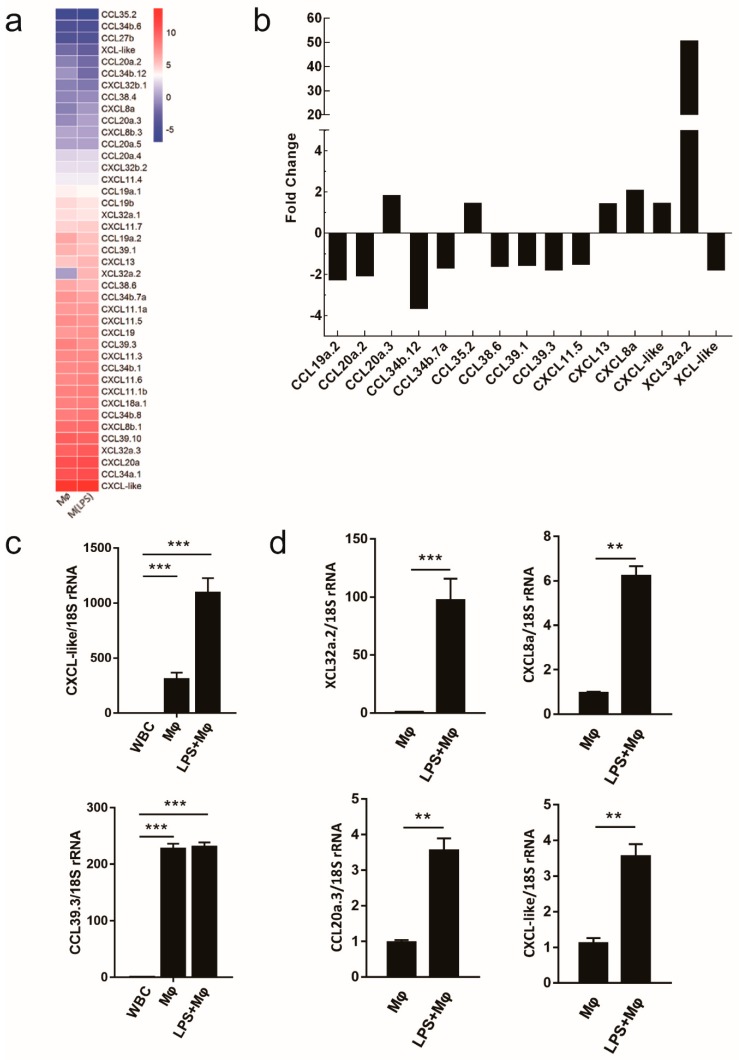
Evaluation of chemokines as macrophage markers. (**a**) Using WBC as a control, all C, C–C and C–X–C chemokines were selected from the set of regulated transcripts and sorted according to average expression level changes. (**b**) The transcriptome was further screened for the identification of the changes in chemokines during macrophage activation. (**c**) The mRNA expression levels of chemokines in Mø and M(LPS) compared to WBC were tested by qRT-PCR. (**d**) The fold changes of mRNA expression in M(LPS) relative to Mø. Data are expressed as mean ± SD (*n* = 3). * *P* < 0.05; ** *P* < 0.01; *** *P* < 0.001.

Chemokines not only played an important functional role in macrophage activity but also included some of the earliest proposed markers of macrophage activation [33,34]. We generated a heat map of transcript expression changes for chemokines from the C, C–C and C–X–C subfamilies from the transcripts. The chemokine expression profiles of macrophages (Mø and M(LPS)) were significantly different from WBC. Relatively speaking, the expression profiles from Mø, compared to M(LPS), appeared to have more similar trends in chemokine expression (Figure 6a, Supplementary Table S2). In order to further ascertain the phenotype of macrophages, we noted that the bar chart results supported the difference in the gene expression between the resting and activated macrophages (Figure 6b, Supplementary Dataset 2).

qRT-PCR validation transcriptome expression levels for each library experiment showed that the transcripts for two chemokines, *CXCL*-like and *CCL39.3*, accumulated in macrophages (Mø and M(LPS)) (Figure 6c). After LPS induction, the *XCL32a.2*, *CXCL8a*, and *CCL20a.3* were expressed in activated macrophages (Figure 6d). These observations suggested that *CXCL*-like and *CCL39.3* could be used as specific marker-genes for macrophages. Additionally, *XCL32a.2* and *CXCL8a* could be seen as specific marker-genes for activating macrophages. In this part, samples obtained from three independent experiments showed the same trend (Supplementary Dataset 2).

## 3. Discussion

The innate immune system is the first line of defense against pathogenic microorganisms [35]. Macrophages have a high degree of heterogeneity, distribution of different tissues and organs, or changes in the local microenvironment, and even different stimulation of macrophages in vitro will lead to different immune responses and access to different functional phenotypes [10,23]. In recent years, the research on the polarization of fish macrophages gradually goes deeper. However, few studies have focused on the markers of teleosts macrophages. Using a combined transcriptome and qRT-PCR-based approach in this study, the classical markers were used to identify macrophages and novel markers of Mø and M(LPS) were identified.

Microbial stimuli such as LPS from Gram-negative bacteria induced robust metabolic rewiring in immune cells [36]. Specifically, in our work, transcriptome analysis and qRT-PCR analysis of the regulated transcripts each supported the concept that *C. idella* macrophages transcriptome Mø and M(LPS) were situated close to each other with little change observed after stimulation with LPS. Previous studies demonstrated that LPS activated NF-κB and MAPK via TRIF and MyD88 in mammals [32]. Among them, TLR4 plays a critical role in the above process [37,38], it was very likely that fish did not have the exact same immune response to LPS as found in mammals. However, this response still showed characteristics of classically activated macrophages. As *C. idella* pathologic immune responses may be regulated through the other unidentified biological pathways, rather than TLR4 receptor complex, or as previous research has been shown to be associated with contaminants in the LPS preparation [39], the final activation status was still consistent with the M1 profile. Accumulated evidence suggested that transcription factor signal transducer and activator of transcription 1 (STAT1) sponsorship were required to increase iNOS and M1-related cytokines production [8,40]. However, in this study, the up-regulation of iNOS message did not did not alter the production of NO in M(LPS) (Supplementary Figure S6). This might indicate that nitrogen intermediates were not able to be produced by the slight upregulation of iNOS.

According to our initial time course analysis, the previously proposed macrophage markers *CD68* and *M-CSFR* were found to be expressed in both Mø and M(LPS). LPS was used in this study to generate a macrophage pro-inflammatory phenotype from head-kidney macrophages, the predisposing factor in this process was that macrophages might undergo phenotype changes and produce pro-inflammatory markers. Consistent with previous research, LPS treatment increased the mRNA levels of pro-inflammatory factors, including IL-1β, IL-6, TNF-α and iNOS in *C. idella* macrophages, indicating the occurrence of innate inflammatory responses in fish, as seen in mammals [41,42,43]. Meanwhile, after stimulation with LPS, we observed an increase of respiratory burst activity in macrophages (Figure 2c). Therefore, a transcriptome analysis was used to identify the potential markers of different phenotype macrophages.

Communication among cells and between cells and their environment is realized through the plasma membrane. Membrane receptors play a crucial role in the exchange of information between cells and the environment. Furthermore, numerous subpopulations of macrophages are defined by the presence or absence of multiple cell surface markers, that is, their stages of differentiation are uniquely characterized by different expression levels of multiple cell surface markers known as clusters of differentiation. For example, in mammals, a classically activated (M1) macrophage phenotype has been found to be associated with the expression of *CD80* and *CD86* [44,45], alternatively activated (M2) macrophage is phenotypically characterized by the expression of specific markers *CD14*, *CD163* and *CD206* [46].

In this investigation, we found that Mø and M(LPS) produced significant levels of *CD63*, *CD48*, *CD276*, especially *CD22*-like at the mRNA level. Lots of studies have shown that *CD22* is a surface marker of lymphocytes [47,48]. In the current study, we determined that *CD22*-like abundantly expressed in resting and activated macrophages. In mammals, it has been shown that tetraspanins were expressed differentially in monocyte subsets, with *CD63* exhibiting higher expression level; what is more, *CD63* could bind with typhimurium, which confirmed its importance [49]. We speculate that *CD63* also plays an important role in bacterial infection in *C. idella*. Studies have shown that the cell-surface receptor CD48 was a lipid-anchored protein expressed on all antigen-presenting cells and T cells [50] and the research by Möller suggests that specific contacts between the macrophages and *E. coli* were formed via the glycoprotein CD48 on filopodia and the adhesin FimH on type 1 fimbriae (hook) [51]. *CD276* acted as both a T cell costimulator and coinhibitor that played a potent role in T cell responses. Previous research found that *CD276* promoted TNF-α secretion [48]. Whether *CD276* exerts the same functions in the immune system of *C. idella* remains unraveled. When macrophages were activated by the treatment with LPS, the mRNA expression level of *CD69* was increased. In addition to *CD69*, activated macrophages also expressed *CD180* and *CD27*. *CD69* has been demonstrated to be a surface marker of LPS-induced macrophages in mammals [52]. Several studies have confirmed the increased expression of *CD180* enabled the intensive phagocytic function of monocytes/macrophages and secretion of proinflammatory cytokines [53,54]. According to our results, we hypothesized that *CD27* may also serve as a possible surface marker for such cells.

It is well known that LPS activation of monocytes/macrophages leads to the production of cytokines, chemokines and toxic mediators [55]. Additionally, chemokine receptors and ligands are differentially modulated in polarized macrophages. In this study, a variety of chemokines have been detected in both Mø and M(LPS) as the products of macrophages or matrix components including *CXCL*-like and *CCL*39.3. Besides, macrophage inflammatory proteins (a special chemokine XCL32a.2, CXCL8a (IL-8) and CCL20a.3) were produced by macrophages after stimulation with LPS, we speculate that these chemokines may play a crucial role in the immune response [44]. The LPS-induced cytokine production and chemotactic factors expression were predominantly characterized by inhibited various PRRs-mediated NF-κB signaling pathways [56]. In our study, *C. idella* may or may not induce an immune response through the NF-κB pathway on a species dependent basis, which is yet to be studied. Growing evidence has suggested that *CXCL8a* (IL-8) is critical in melanoma progression and in the upregulation of biological responses in that it can stimulate neoplastic growth, promote inflammation and induce angiogenesis [57]. The functions of *XCL32a.2* need to be further analyzed.

Previously published macrophage markers were tested for validation. Transcriptome sequencing analysis and qRT-PCR method (three biological replicates) were applied to screen the marker genes of different phenotypic macrophages. In summary, measuring the expression changes of well-characterized markers would provide valuable proof to accurately differentiate various activation states associated with the functional activity of macrophages populations in teleosts.

## 4. Materials and Methods

### 4.1. Ethical Statement

The recommendations about the Guide for the Care and Use of Laboratory Animals of the National Institutes of Health were strictly carried out in this research. The use of experimental fish was approved by the Animal Ethics Committee of Huazhong Agricultural University on 15 March 2017. The tissue material from fish was used for the present study did not involve endangered or protected species. All experimental animals were anesthetized with 3-Aminobenzoic acid ethyl ester methanesulfonate (MS-222), and every effort was made to minimize suffering. The reporting of this study adheres to the ARRIVE Guidelines for reporting animal research. A completed ARRIVE guidelines checklist was included in Additional file 1: ARRIVE checklist.

### 4.2. Fish Rearing

*C. idella* specimens (*n* = 100, average weight = 500 ± 50 g) were obtained from Hubei Bairong Improved Aquatic Seed Co, Ltd. (Huanggang, China). The fish were distributed into four tanks (~25 per tank) and acclimated for a month. During this period, the water temperature of the fish tanks was kept at 20  ±  5 °C, and the fish were fed twice daily with a commercial fish feed (Haid Group, China) throughout the experiments.

### 4.3. Isolation of Head-Kidney Leukocytes and Macrophages

Head-kidney leukocytes were obtained using a partly modified method previously reported [58]. Briefly, ten fish from each group were anesthetized with 0.02% 3-Aminobenzoic acid ethyl ester methanesulfonate (MS-222), head-kidneys were removed aseptically and then let them pass through a 100 μm mesh in Leibovitz medium (L-15). A cell suspension was layered on a 51% Percoll gradient and then centrifuged at 300× *g* for 30 min at 4 °C. After this, the middle layer of cells was collected and then layered onto a discontinuous (34%/51%) Percoll density gradient and centrifuged at 400× *g* for 30 min at 4 °C. The band lying at the interface was collected and washed twice with L-15 medium. Subsequently, the cells were randomly split into two groups, one of which was used to obtain leukocytes (WBC). The other group of cells was cultivated in 2 mL L-15 (Boster Biological Technology, Wuhan, China) supplemented with 2% fetal bovine serum (FBS), 50 µg/mL gentamicin for 4 h and seeded into two six-well plate at a density of 2 × 10^6^ cells per well. In this way, highly pure macrophages can be obtained. A portion of the cells was cultured for an additional 6 h in 2 mL L-15 with 10% FCS to obtain resting macrophages (Mø). To obtain activated phenotype (M(LPS)), the remaining macrophages were stimulated with 5 μg/mL LPS at the same time under the same conditions. In simple terms, three sets of samples were obtained: freshly isolated leukocytes (WBC), resting macrophages (Mø) and activated macrophages (M(LPS)). All cells were identified by Giemsa staining and ultrastructural observations [41].

### 4.4. Respiratory Burst Activity

Before and after stimulation with LPS, macrophages respiratory burst activity was analyzed with the nitroblue tetrazolium (NBT) assay. Macrophages were incubated at 28 °C for 60 min in 100 μL of NBT (Biofroxx, Guanzhou, China) solution (1 mg/mL in sterile HBSS) with 1 μg/mL of phorbol myristate acetate (PMA) (Yesen, Shanghai, China). After that, the reaction was stopped with 80% methanol, the cells were washed and air-dried before the addition of 140 μL of dimethyl sulfoxide (DMSO) and 120 μL of 2 M KOH, the absorbance was read at 620 nm.

### 4.5. RNA Extraction and RNA Sequencing by Illumina Hiseq

Total cellular RNA was extracted using TRIzol Reagent (Invitrogen, Carlsbad, CA, USA) according to the manufacturer’s protocol. RNA samples were quantified and quality checked by Agilent 2100 Bioanalyzer (Agilent Technologies, Palo Alto, CA, USA), and 1% agarose gel. 1 μg of total RNA (RIN value above 7) from each sample was used for RNA sequencing analysis. Next, the sequencing library was constructed according to the manufacturer’s protocol (NEBNext^®^ Ultra™ RNA Library Prep Kit for Illumina^®^, Beijing, China).

The poly(A) mRNA isolation was performed using NEBNext Poly(A) mRNA Magnetic Isolation Module (NEB). The mRNA fragmentation and priming were performed using NEBNext First Strand Synthesis Reaction Buffer and NEBNext Random Primers. First strand cDNA synthesis was performed with ProtoScript II Reverse Transcriptase and second strand synthesis with Second Strand Synthesis Enzyme Mix. After that, the double-stranded cDNA was purified using Axygen AxyPrep Mag PCR clean-up kit. Samples were then treated with End Prep Enzyme Mix to repair the ends, A-tails were added to the sheared ends. Adapter-ligated DNA was then purified using AxyPrep Mag PCR Clean-up (Axygen, Shanghai, China), and the fragments in the range of 300 to 360 bp were selected. Each sample was heat inactivated and subsequently amplified by PCR for 11 cycles using P5 and P7 primers. These two primers carry a sequence that can be annealed to “bridged PCR” and P7 primers carrying a six-base index. After each step, the PCR products were cleaned up using commercial PCR product purification kit (Axygen), validated using an Agilent 2100 Bioanalyzer and the final concentration was measured by Qubit 2.0 Fluorometer (Invitrogen, Carlsbad, CA, USA). Finally, the sample was sequenced using Illumina HiSeq 2000 platforms at the Beijing Genome Institute (BGI).

### 4.6. Differential Gene Expression Analysis

In order to improve the quality of the data, we removed technical sequences to get high-quality clean data. Differential expression analysis was performed using the DESeq Bioconductor package based on the negative binomial distribution. After adjusting by Benjamini and Hochberg’s approach to control the false discovery rate, the absolute value of log2 Ratio  ≥  1 and *P* ≤ 0.05 were used to detect differential expressed ones. Differentially expressed genes were investigated using the RPKM method (reads per kilo bases per million reads).

Differentially expressed probe sets were analyzed for Gene Ontology (GO) enrichment and selected using a *P*-value less than 0.05 [59]. Differentially expressed genes were mapped to the KEGG database (available online: http://en.wikipedia.org/wiki/KEGG) and identified significantly enriched KEGG terms. Principal component analysis (PCA) was another way to visualize sample-to-sample distances. In this ordination method, the samples were projected onto the 2D plane and they spread out in the two directions, the variation reflected by the two directions explained most of the differences. The x-axis was the direction that separated the data points the most. The values of the samples in this direction were written in PC1. The y-axis was the direction (it must be orthogonal to the first direction) that separated the data the second most. The values of the samples in this direction were written in PC2. The percent of the total variance that was contained in the direction was printed in the axis label. Note that these percentages did not add to 100%, because there were more dimensions that contained the remaining variance (although each of these remaining dimensions would explain less than the two dimensions do). This analysis has been processed with R language.

### 4.7. Confirmation of Gene Expression Profiles by qRT-PCR

To confirm the gene expression pattern of highly up- or down-regulated DEGs, qRT-PCR was performed using a Roche LightCycler^®^ 480 system and the 2×T5 Fast qPCR Mix (SYBR Green I) (Beijing TsingKe Biotech Co., Ltd., Wuhan, China). Three independent biological replicates and three technical replicates of each biological replicate were used for real-time PCR analysis. Briefly, 30 individuals were randomly divided into 3 groups to isolate and purify macrophages at different times. Additionally, each sample was collected from six wells of a six-well plate in 10 individuals and measurement was repeated 3 times. The housekeeping gene 18S rRNA served as an internal control. qRT-PCR and data analysis were performed according to the protocol and method as described previously [60]. Sequence-specific primers were designed using Premier Primer 5 software listed in (Table 2). Unpaired Student’s *t*-test was used in the data analysis, and the *P*-value less than 0.05 was considered as a statistically significant difference (* *P* < 0.05, ** *P* < 0.01, ** *P* < 0.001).

## 5. Conclusions

Macrophages play a critical role in host defence, wound healing and immune regulation. Using a combined transcriptome- and qRT-PCR- based approach in this study, the previously proposed markers of macrophages were better characterized and novel markers of Mø and M(LPS) were identified. Our results suggested that four novel cell surface marker genes (CD22-like, CD63, CD48 and CD276) and two chemokine genes (CXCL-like and CCL39.3) would be emerging potential marker genes of macrophage in grass carp. Furthermore, CD69, CD180, CD27, XCL32a.2 and CXCL8a genes can be used as marker genes to confirm whether macrophages are activated. Measuring the expression changes of well-characterized markers would provide a valuable proof to accurately differentiate various activation states associated with functional activity of macrophages populations in teleosts. And these novel marker genes may represent a potential target for macrophages activation in teleosts.

## Figures and Tables

**Table 1:** Summary of the transcriptome data of three kinds of cells.

Transcriptome Analysis Statistics	WBC	Mø	M(LPS)
MiSeq statistics			
Raw reads	55,358,880	42,691,264	54,911,804
Average read length (bp)	150	150	150
Total base pairs (bp)	8,303,832,000	6,403,689,600	8,236,770,600
≥ Q20 of clean reads (%)	91.42	91.8	92.8
Clean reads	53,792,212	41,600,582	53,849,176
Average read length (bp)	146	146	147
Total base pairs (bp)	7,871,999,232	6,089,281,739	7,904,942,691
≥ Q20 of clean reads (%)	93.39	93.64	94.36
Mapping (Analysis of comparison of clean data with reference genomes of *C. idella*)			
Total mapped (%)	85.16	85.73	86.86
Multiple mapped (%)	4.63	4.51	4.49
Uniquely mapped (%)	80.53	81.22	82.36
Reads map to ‘+’	21,615,571	16,841,994	22,110,713
Reads map to ‘−’	21,706,658	16,946,649	22,243,457
Non_splice reads	25,894,935	18,912,591	24,597,501
Splice reads	17,427,294	14,876,052	19,756,669
Reads mapped in proper pairs	38,542,714	30,245,418	40,189,646

**Table 2:** Primers used in the experiment.

Gene Number	Gene	Primer Name	Forward Primer (5′→3′)	Primer Name	Reverse Primer (5′→3′)
EU047719	18S rRNA	18F99	ATTTCCGACAC GGAGAGG	18R100	CATGGGTTTAGGATACGCTC
KF444352.1	M-CSFR	M-CSFR-F	CGCTCGACATGGACGACTTA	M-CSFR-R	ACTCGACTGTTGGTGAGCAG
JQ040498.1	TNF-α	TNF-α-F	GCTGCTGTCTGCTTCACGC	TNF-α-R	AGCCTGGTCCTGGTTCACTCT
JQ692172.1	IL-1β	IL-1β-F	TTGGAAATGTGGAGGC ATTCT	IL-1β-R	GATGTTGAGCACCTCTTCTTCA
KC535507.1	IL-6	IL-6-F	CTCAACCCTGGTCAACGACA	IL-6-R	GCATCCATGCGGATTTGACC
HQ589354.1	iNOS	iNOS-F	TTCACATGGAGCACCCACAA	iNOS-R	TCAGTGCCCATGTACCAACC
JN255694	CXCL8a	CXCL8a-F	TCGTTGGCAGAATGAACTGC	CXCL8a-R	TAATGCAGCGACAGCGTAG
MF783120*	CCL39.3	CCL39.3-F	GAAGCCTGATGTTTCTGCTGG	CCL39.3-R	AGACTTCACCAGTTTCACAGGAA
MF783159*	CXCL-like	CXCL-like-F	GTTTGCTGGCTGTCAACCTC	CXCL-like-R	AGCAGTTAGGTCCTTTTGGAGT
MF783144*	XCL32a.2	XCL32a.2-F	TCCTCACCATGGGCTGTTAAT	XCL32a.2-R	TTTGACATTTTCTTAGGAGCCGC
MF783098*	CCL20a.3	CCL20a.3-F	TCGTGATCGTGCTGATGGTT	CCL20a.3-R	GATGTGGCAGTTTCTTGTCATGT
CI01000344_01208746_01214936	CD68	CD68-F	TATGGGGAACGGTGTGAGTC	CD68-R	CGATAGCGACACGGTAGTCA
CI01000001_13329841_13334948	CD63	CD63-F	TTGGAGCTTTGGGAGATGACA	CD63-R	TGATCTGCACAAACGCGATG
CI01000343_00067132_00076892	CD48	CD48-F	CGTGGCCCTTAATTGCACTG	CD48-R	CGAAGAGTCGACAGCATTTCC
CI01000219_00233215_00238585	CD22-like	CD22-like-F	CAGCTCTGACGGAAACCACT	CD22-like-R	CAGCTCTGACGGAAACCACT
CI01000017_00722020_00762518	CD276	CD276-F	GGCTCTGTTTGGGATGGACA	CD276-R	GGCTCTGCCGATAACTGTGTA
CI01000059_00691603_00693842	CD180	CD180-F	TGTCCACCTTGTCCCATCTG	CD180-R	CTGAATCCGAGTTGCTTCGC
CI01000365_00126423_00134788	CD11-1	CD11-1-F	CGATCTCAACGCTCAGAAAGC	CD11-1-R	ACCCAATGATGTAGCGGAGG
CI01000365_00275086_00325578	CD11-2	CD11-2-F	TCGGGTCATATTTCGGAGCG	CD11-2-R	AGACGTACAGCCTCCCTTCT
CI01000027_02147653_02152768	CD22	CD22-F	AATATACTGGACCGGGTGGC	CD22-R	TCCGGGGTACAGTTTTCTGTG
CI01092720_00000024_00000324	CD69	CD69-F	CAACATGAACGACACGAACGA	CD69-R	ACCTGAAGACCACTGCCATTT
CI01000057_01361967_01364527	CD27	CD27-F	TATTTGTGGGGGCGCTTAGT	CD27-R	TCGGAGCAGTTCTTGGTAACAT

* indicates the sequence was deposited in GenBank by ourselves.

## Data Availability

The datasets generated and/or analysed during the current study are publicly available at NCBI–SRA (www.ncbi.nlm.nih.gov/sra) accession: SRR6683542, SRR6683539 and SRR6683538.

## Supplementary Materials

Supplementary materials can be found at http://www.mdpi.com/1422-0067/19/11/3562/s1.

## References

[1] Hirayama D., Iida T., Nakase H. (2017). The Phagocytic Function of Macrophage-Enforcing Innate Immunity and Tissue Homeostasis. Int. J. Mol. Sci..

[2] Hume D.A., Allan W., Fabrus B., Weidemann M.J., Hapel A.J., Bartelmez S. (1987). Regulation of proliferation of bone marrow-derived macrophages. Lymphokine Res..

[3] McDonald L., Mehrotra M., LaRue A. (2015). Hematopoietic origin of murine lung fibroblasts. Stem Cells Int..

[4] Chaves-Pozo E., Mulero V., Meseguer J., Ayala A.G. (2005). Professional phagocytic granulocytes of the bony fish gilthead seabream display functional adaptation to testicular microenvironment. J. Leukoc. Biol..

[5] Lu J.W., Hsieh M.S., Liao H.A., Yang Y.J., Ho Y.J., Lin L.I. (2015). Zebrafish as a Model for the Study of Human Myeloid Malignancies. Biomed Res. Int..

[6] Lu X.J., Chen Q., Rong Y.J., Chen F., Chen J. (2017). CXCR3.1 and CXCR3.2 Differentially Contribute to Macrophage Polarization in Teleost Fish. J. Immunol..

[7] Zhou L.N., Bi C.S., Gao L.N., An Y., Chen F., Chen F.M. (2018). Macrophage polarization in human gingival tissue in response to periodontal disease. Oral Dis..

[8] Murray P.J. (2017). Macrophage Polarization. Annu. Rev. Physiol..

[9] Gordon S. (2003). Alternative activation of macrophages. Nat. Rev. Immunol..

[10] Taylor P., Martinez-Pomares L., Stacey M., Lin H., Brown G., Gordon S. (2005). Macrophage receptors and immune recognition. Annu. Rev. Immunol..

[11] Mills C.D., Kincaid K., Alt J.M., Heilman M.J., Hill A.M. (2017). M-1/M-2 macrophages and the Th1/Th2 paradigm. J. Immunol..

[12] Hume D.A. (2015). The many alternative faces of macrophage activation. Front. Immunol..

[13] Kollipara R.K., Perumal N.B. (2010). Motif prediction to distinguish LPS-stimulated pro-inflammatory vs. antibacterial macrophage genes. Immunome Res..

[14] Ng W., Marinov G., Chin Y., Lim Y., Ea C. (2017). Transcriptomic analysis of the role of RasGEF1B circular RNA in the TLR4/LPS pathway. Sci. Rep..

[15] Menchetti L., Barbato O., Filipescu I., Traina G., Leonardi L., Polisca A., Troisi A., Guelfi G., Piro F., Brecchia G. (2017). Effects of local lipopolysaccharide administration on the expression of Toll-like receptor 4 and pro-inflammatory cytokines in uterus and oviduct of rabbit does. Theriogenology.

[16] Fong C.H.Y., Bebien M., Didierlaurent A., Nebauer R., Hussell T., Broide D., Karin M., Lawrence T. (2008). An antiinflammatory role for IKK beta through the inhibition of “classical” macrophage activation. J. Exp. Med..

[17] Nair M.G., Du Y., Perrigoue J.G., Zaph C., Taylor J.J., Goldschmidt M., Swain G.P., Yancopoulos G.D., Valenzuela D.M., Murphy A. (2009). Alternatively activated macrophage-derived RELM-alpha is a negative regulator of type 2 inflammation in the lung. J. Exp. Med..

[18] El Kasmi K.C., Qualls J.E., Pesce J.T., Smith A.M., Thompson R.W., Henao-Tamayo M., Basaraba R.J., Koenig T., Schleicher U., Koo M.-S. (2008). Toll-like receptor-induced arginase 1 in macrophages thwarts effective immunity against intracellular pathogens. Nat. Immunol..

[19] Rigamonti E., Zordan P., Sciorati C., Rovere-Querini P., Brunelli S. (2014). Macrophage plasticity in skeletal muscle repair. Biomed Res. Int..

[20] Kreider T., Anthony R.M., Urban J.F., Gause W.C. (2007). Alternatively activated macrophages in helminth infections. Curr. Opin. Immunol..

[21] Anthony R.M., Urban J.F., Alem F., Hamed H.A., Rozo C.T., Boucher J.-L., van Rooijen N., Gause W.C. (2006). Memory T(H)2 cells induce alternatively activated macrophages to mediate protection against nematode parasites. Nat. Med..

[22] Martinez F.O., Helming L., Gordon S. (2009). Alternative Activation of Macrophages: An Immunologic Functional Perspective. Annu. Rev. Immunol..

[23] Stout R.D., Jiang C.C., Matta B., Tietzel I., Watkins S.K., Suttles J. (2005). Macrophages sequentially change their functional phenotype in response to changes in microenvironmental influences. J. Immunol..

[24] Stout R.D., Suttles J. (2004). Functional plasticity of macrophages: Reversible adaptation to changing microenvironments. J. Leukoc. Biol..

[25] Bao C., Wang B., Yang F., Chen L. (2018). Blockade of Interleukin-7 Receptor Shapes Macrophage Alternative Activation and Promotes Functional Recovery After Spinal Cord Injury. Neuroscience.

[26] Barreda D., Belosevic M. (2001). Transcriptional regulation of hemopoiesis. Dev. Comp. Immunol..

[27] Murray P., Allen J., Biswas S., Fisher E., Gilroy D., Goerdt S., Gordon S., Hamilton J., Ivashkiv L., Lawrence T. (2014). Macrophage activation and polarization: Nomenclature and experimental guidelines. Immunity.

[28] Jiang H., Hu Y., Wei X., Xiao X., Jakovlic I., Liu X., Su J., Yuan G. (2018). Chemotactic effect of beta-defensin 1 on macrophages in Megalobrama arnblycephala. Fish Shellfish Immunol..

[29] Chen Q., Lu X., Li M., Chen J. (2016). Molecular cloning, pathologically-correlated expression and functional characterization of the colonystimulating factor 1 receptor (CSF-1R) gene from a teleost, *Plecoglossus altivelis*. Zool. Res..

[30] Deng F., Chen W., Liu L., Wang L., Chen X. (2012). The expression and molecular mechanism of M1 macrophages in rheumatic valvular disease. Zhonghua Wai Ke Za Zhi.

[31] Wiegertjes G.F., Wentzel A.S., Spaink H.P., Elks P.M., Fink I.R. (2016). Polarization of immune responses in fish: The ‘macrophages first’ point of view. Mol. Immunol..

[32] Sudan B., Wacker M.A., Wilson M.E., Graff J.W. (2015). A systematic approach to identify markers of distinctly activated human macrophages. Front. Immunol..

[33] Mantovani A., Sica A., Sozzani S., Allavena P., Vecchi A., Locati M. (2004). The chemokine system in diverse forms of macrophage activation and polarization. Trends Immunol..

[34] Liao Z.W., Wan Q.Y., Xiao X., Ji J.F., Su J.G. (2018). A systematic investigation on the composition, evolution and expression characteristics of chemokine superfamily in grass carp Ctenopharyngodon Idella. Dev. Comp. Immunol..

[35] Holden J.A., Attard T.J., Laughton K.M., Mansell A., O’Brien-Simpson N.M., Reynolds E.C. (2014). *Porphyromonas gingivalis* lipopolysaccharide weakly activates M1 and M2 polarized mouse macrophages but induces inflammatory cytokines. Infect. Immun..

[36] Lachmandas E., Boutens L., Ratter J., Hijmans A., Hooiveld G., Joosten L., Rodenburg R., Fransen J., Houtkooper R., van Crevel R. (2016). Microbial stimulation of different Toll-like receptor signalling pathways induces diverse metabolic programmes in human monocytes. Nat. Microbiol..

[37] Sepulcre M.P., Alcaraz-Perez F., Lopez-Munoz A., Roca F.J., Meseguer J., Cayuela M.L., Mulero V. (2009). Evolution of lipopolysaccharide (LPS) recognition and signaling: Fish TLR4 does not recognize LPS and negatively regulates NF-kappaB activation. J. Immunol..

[38] Holen E., Lie K.K., Araujo P., Olsvik P.A. (2012). Pathogen recognition and mechanisms in Atlantic cod (*Gadus morhua*) head kidney cells Bacteria (LPS) and virus (poly I:C) signals through different pathways and affect distinct genes. Fish Shellfish Immunol..

[39] Seppola M., Mikkelsen H., Johansen A., Steiro K., Myrnes B., Nilsen I.W. (2015). Ultrapure LPS induces inflammatory and antibacterial responses attenuated in vitro by exogenous sera in Atlantic cod and Atlantic salmon. Fish Shellfish Immunol..

[40] Gan Z., Wang Q., Li J., Wang X., Wang Y., Du H. (2017). Iron reduces M1 macrophage polarization in RAW264.7 macrophages associated with inhibition of STAT1. Mediat. Inflamm..

[41] Liu L., Zhou Y., Zhao X., Wang H., Wang L., Yuan G., Asim M., Wang W., Zeng L., Liu X. (2014). Oligochitosan stimulated phagocytic activity of macrophages from blunt snout bream (*Megalobrama amblycephala*) associated with respiratory burst coupled with nitric oxide production. Dev. Comp. Immunol..

[42] Liu X., Li J., Peng X., Lv B., Wang P., Zhao X., Yu B. (2016). Geraniin inhibits LPS-induced THP-1 macrophages switching to M1 phenotype via SOCS1/NF-κB pathway. Inflammation.

[43] Wei H., Yin L., Feng S., Wang X., Yang K., Zhang A., Zhou H. (2015). Dual-parallel inhibition of IL-10 and TGF-β1 controls LPS-induced inflammatory response via NF-κB signaling in grass carp monocytes/macrophages. Fish Shellfish Immunol..

[44] Kalish S., Lyamina S., Usanova E., Manukhina E., Larionov N., Malyshev I. (2015). Macrophages reprogrammed in vitro towards the M1 phenotype and activated with LPS extend lifespan of mice with ehrlich ascites carcinoma. Med. Sci. Monit. Basic Res..

[45] Lee C., Guo Y., So K., Vijayan M., Guo Y., Wong V., Yao Y., Lee K., Chiu P., Yeung W. (2015). Soluble human leukocyte antigen G5 polarizes differentiation of macrophages toward a decidual macrophage-like phenotype. Hum. Reprod..

[46] Soldano S., Pizzorni C., Paolino S., Trombetta A., Montagna P., Brizzolara R., Ruaro B., Sulli A., Cutolo M. (2016). Alternatively activated (M2) macrophage phenotype is inducible by endothelin-1 in cultured human macrophages. PLoS ONE.

[47] Rossi E., Goldenberg D., Michel R., Rossi D., Wallace D., Chang C. (2013). Trogocytosis of multiple B-cell surface markers by CD22 targeting with epratuzumab. Blood.

[48] Bachanova V., Frankel A., Cao Q., Lewis D., Grzywacz B., Verneris M., Ustun C., Lazaryan A., McClune B., Warlick E. (2015). Phase I study of a bispecific ligand-directed toxin targeting CD22 and CD19 (DT2219) for refractory B-cell malignancies. Clin. Cancer Res..

[49] Hassuna N., Monk P., Ali F., Read R., Partridge L. (2017). A role for the tetraspanin proteins in Salmonella infection of human macrophages. J. Infect..

[50] Abadía-Molina A., Ji H., Faubion W., Julien A., Latchman Y., Yagita H., Sharpe A., Bhan A., Terhorst C. (2006). CD48 controls T-cell and antigen-presenting cell functions in experimental colitis. Gastroenterology.

[51] Möller J., Lühmann T., Chabria M., Hall H., Vogel V. (2013). Macrophages lift off surface-bound bacteria using a filopodium-lamellipodium hook-and-shovel mechanism. Sci. Rep..

[52] Ishizaki S., Kasuya Y., Kuroda F., Tanaka K., Tsuyusaki J., Yamauchi K., Matsunaga H., Iwamura C., Nakayama T., Tatsumi K. (2012). Role of CD69 in acute lung injury. Life Sci..

[53] Tsertsvadze T., Mitskevich N., Bilanishvili A., Girdaladze D., Porakishvili N. (2017). Phagocytosis and expression of FCg-receptors and CD180 on monocytes in chronic lymphocytic leukemia. Georgian Med. News.

[54] Yu C., Micaroni M., Puyskens A., Schultz T., Yeo J., Stanley A., Lucas M., Kurihara J., Dobos K., Stow J. (2015). RP105 engages phosphatidylinositol 3-kinase p110δ to facilitate the trafficking and secretion of cytokines in macrophages during mycobacterial infection. J. Immunol..

[55] Lo D., Feng L., Li L., Carson M., Crowley M., Pauza M., Nguyen A., Reilly C. (1999). Integrating innate and adaptive immunity in the whole animal. Immunol. Rev..

[56] Lei Q., Li L., Cai J., Huang W., Qin B., Zhang S. (2016). ORF3 of hepatitis E Virus inhibits the expression of proinflammatory cytokines and chemotactic factors in LPS-stimulated human PMA-THP1 cells by inhibiting NF-κB pathway. Viral Immunol..

[57] Haghnegahdar H., Du J., Wang D., Strieter R.M., Burdick M.D., Nanney L.B., Cardwell N., Luan J., Shattuck-Brandt R., Richmond A. (2000). The tumorigenic and angiogenic effects of MGSA/GRO proteins in melanoma. J. Leukoc. Biol..

[58] Davidson G., Lin S., Secombes C., Ellis A. (1997). Detection of specific and ‘constitutive’ antibody secreting cells in the gills, head kidney and peripheral blood leucocytes of dab (*Limanda limanda*). Vet. Immunol. Immunopathol..

[59] Bai Z., Yin Y., Hu S., Wang G., Zhang X., Li J. (2009). Identification of genes involved in immune response, microsatellite, and SNP markers from expressed sequence tags generated from hemocytes of freshwater pearl mussel (*Hyriopsis cumingii*). Mar. Biotechnol..

[60] Wan Q., Su J. (2015). Transcriptome analysis provides insights into the regulatory function of alternative splicing in antiviral immunity in grass carp (*Ctenopharyngodon idella*). Sci. Rep..

