# Supplementary material for: Transcriptome Analysis Provides Insights into the Markers of Resting and LPS-Activated Macrophages in Grass Carp (Ctenopharyngodon idella)
Source: Int J Mol Sci. 2018 Nov 12;19(11):3562. doi: 10.3390/ijms19113562 (PMC6274997; doi:10.3390/ijms19113562)
Supplement: Supplementary file 1 [file ijms-19-03562-s001.zip › ijms-378862-supplementary.doc]

**Transcriptome analysis provides insights into the markers of resting and LPS-activated macrophages in grass carp (*Ctenopharyngodon idella*)**

**Yazhen Hua,b, Xiaolei Weia,b, Zhiwei Liaoa,b, Yu Gaoc, Xiaoling Liua,b, Jianguo Sua,b, Gailing Yuana,b,***

*^a^* *Department of Aquatic Animal Medicine, College of Fisheries,* *Huazhong Agricultural University, Wuhan* *430070, China*

*^b^ Hubei Engineering Technology Research Center for Aquatic Animal Disease Control and Prevention*

*^c^ College of Animal Science and Technology, Yunnan Agricultural University, Kunming 650201, China*

**Supplementary Figures**

**Supplementary Figure S1 |** **Verified mRNA expressions of previously proposed markers in transcriptome sequencing****.** **(a)** CD68 and M-CSFR as the classical surface markers of macrophage were determined in Mø and M(LPS). **(b)** Proinflammatory factor levels of IL-1β, IL-6, iNOS (which elicited the production of reactive nitrogen), and TNF-α were determined in macrophage culture in vitro, stimulated with LPS (5μg/mL) or without stimulation. **P* < 0.05; ***P* < 0.01; ****P* < 0.01.

**Supplementary Figure S2 | All the three independent experiments had the same trend on previously reported markers of macrophage by qRT-PCR.** **(a)** CD68 and M-CSFR as the classical surface markers of macrophage were determined in Mø and M(LPS). **(b)** Proinflammatory factor levels of IL-1β, IL-6, iNOS, and TNF-α were determined in macrophage culture in vitro, stimulated with LPS (5μg/mL) or without stimulation. Data were expressed as mean ± SD, n=3 due to three technical replicates.

**Supplementary Figure S3 | RPKM density distribution.** The log10(RPKM) is used for the Y axis and the sample name is used for the X axis. Box plots show the median, the lower quartile and the upper quartile, and the lowest and highest values in the distribution of a given set of data.

**Supplementary Figure S4 |** **All the three independent experiments had the same trend on CD molecules by qRT-PCR.** **(a)** CD molecules were expressed in resting and activated macrophages. **(b)** The changes in CD molecule during macrophage polarization. Data were expressed as mean ± SD, n=3 due to three technical replicates.

**Supplementary Figure S5 | All the three independent experiments had the same trend on chemokines by qRT-PCR.** **(a)** Chemokines were expressed in resting and activated macrophages. **(b)** The changes in chemokines during macrophage polarization. Data were expressed as mean ± SD, n=3 due to three technical replicates. * *P* < 0.05; ** *P* < 0.01; *** *P* < 0.01.

**Supplementary Figure S6 | Nitric oxide (NO) production of macrophages.** Macrophages were incubated with 5 μg/mL of LPS or medium alone for 6 h. The nitric oxide (NO) content was measured by using a nitric oxide assay kit. Data were expressed as mean ± SD (n = 3).
